# Atypical sphingosine-1-phosphate metabolites—biological implications of alkyl chain length

**DOI:** 10.1007/s00424-024-03018-8

**Published:** 2024-09-19

**Authors:** Melanie Glueck, Alexandra Lucaciu, Julien Subburayalu, Roxane Isabelle Kestner, Waltraud Pfeilschifter, Rajkumar Vutukuri, Josef Pfeilschifter

**Affiliations:** 1 Institute of General Pharmacology and Toxicology, Pharmazentrum Frankfurt, Goethe University, Frankfurt am Main, 60596 Frankfurt, Germany; 2 Department of Neurology, University Hospital Frankfurt, Frankfurt, Goethe University, Frankfurt am Main, 60528 Frankfurt, Germany; 3 Department of Internal Medicine, University Hospital Carl Gustav Carus TU Dresden, Fetscherstraße 74, 01307 Dresden, Saxony Germany; 4 Center of Regenerative Therapies Dresden, TU Dresden, Fetscherstraße 74, 01307 Dresden, Saxony Germany; 5 Department of Neurology and Clinical Neurophysiology, Städtisches Klinikum Lüneburg, 21339 Lüneburg, Germany; 6 Institute for Transfusion Medicine and Immunohaematology, German Red Cross Blood Donor Service Baden-Württemberg-Hessen, Goethe University Hospital, 60528 Frankfurt Am Main, Germany

**Keywords:** Sphingolipids, Sphingosine-1-phosphate (S1P), Chain lengths, Myristoyl CoA, Palmitoyl CoA, Stearoyl CoA, Serine palmitoyl transferase

## Abstract

Sphingosine-1-phosphate (S1P) is a bioactive lipid signaling molecule with pleiotropic implications by both auto- and paracrine signaling. Signaling occurs by engaging five G protein-coupled receptors (S1P_1-5_) or intracellular pathways. While the extensively studied S1P with a chain length of 18 carbon atoms (d18:1 S1P) affects lymphocyte trafficking, immune cell survival and inflammatory responses, the biological implication of atypical S1Ps such as d16:1 or d20:1 remains elusive. As S1P lipids have far-reaching implications in health and disease states in mammalian organisms, the previous contrasting results may be attributed to differences in S1P’s alkyl chain length. Current research is beginning to appreciate these less abundant atypical S1P moieties. This review provides an up-to-date foundation of recent findings on the biological implications of atypical S1P chain lengths and offers a perspective on future research endeavors on S1P alkyl chain length–influenced signaling and its implications for drug discovery.

## Introduction

Sphingolipids comprise a group of lipid mediators with indispensable structural and signaling functions. The dawn of sphingolipid biology began after seminal experiments by Ludwig Thudicum in 1874, where he first isolated sphingosine from ethanolic extracts of the brain [1]. In 1947, sphingosine was structurally characterized by Carter et al. [7]. In vertebrates, sphingolipids are the second most abundant membrane lipids after glycerol-phospholipids [39]. Structurally, sphingolipids consist of a sphingoid base and a ceramide tail with an acylated fatty acid long chain base (LCB) contributing to their structural diversity [22]. Sphingolipids are bioactive compounds with multiple roles in health and diseases. Especially in the last three decades, pioneering works have established sphingolipids as pivotal signaling molecules in growth, inflammation, vascular integrity, cell survival and cancer [2, 25, 28, 47, 51]. A major breakthrough in acknowledging sphingolipids as therapeutic targets was driven by the discovery of fingolimod, a sphingosine analogue and functional antagonist to the S1P receptor type 1 (S1P_1_). Since its approval by the FDA to treat multiple sclerosis (MS) [5], clinical trials and further drug discoveries to target the sphingolipid pathway, have immensely increased [35]. Although many S1P functions are well-explored, several controversies remain. Discrepancies were often haphazardly attributed to the cell type and environmental stimuli that led to differences in scientific findings. Until recently, this argument, considering the scarcity of available literature, was well-reasoned. However, current research on the differential impact of the alkyl chain length of S1P confers this well-reasoned discussion to question. The occurrence of different sphingosine and sphinganine LCBs is well-described. Nonetheless, only a few studies have explored the functional implications of these varying alkyl chain lengths [21, 34]. In this review, we aim to prime the sphingolipid community for the biological importance of variant alkyl chain lengths in the biology of S1P. We bring together findings of different studies that have focused on the biological implications of these atypical S1P moieties. Emerging genetic and biochemical studies have confirmed the presence of atypical S1P chain lengths. Current research starts to acknowledge their importance in normal and pathological conditions which have significance for the development of S1P signaling-targeting therapeutics.

## Historical and current evidence of atypical sphingolipid chain lengths

After the initial description of sphingolipids in 1947, the following decades have witnessed a tremendous interest in unraveling the manifold biological functions of these enigmatic lipids. Several researchers have reported the presence of LCBs beyond sphingosine in mammalian tissues. For the first time in 1961, the C20-sphingosine was identified by Majhofer-Oreščanin and Proštenik as a component of brain lipids in horse and cow [38]. Stanacev and Chargaff successfully purified C20-sphingosine from the mucolipids of calf brain terming it icosisphingosine [69]. In subsequent studies, C20 LCBs were shown to occur only in gangliosides. Therefore, they were termed gangliosphingosine and dihydrosphingosine (DHS) [61]. Conversely, the presence of C20 sphingosine was validated in sphingomyelin (SM) and cerebrosides. Thus, their contribution as a regular component in the brain and spinal cord of rabbits was confirmed. Interestingly, the group also recorded small amounts of C16 DHS [65]. Using liquid chromatography, the composition of dihydrosphingomyelin (DSM) was described to be 58% of d16:0 LCB and up to 37% of d18:0 LCB origin, whereas sphingomyelin constituted more of d18:1 LCB (57–60%) in comparison to d16:1 LCB (26–30%) [6]. Similarly, the influence of age on sphingolipid homologues was revealed by Rosenberg and Stern where C18 sphingosine was extremely low at birth, which subsequently increased to almost at par levels of C20 sphingosine when aged in brains of rodents [58]. Taken together, all these early studies have established the presence of LCB variants of sphingolipids (C16, C18, and C20) exclusively in the central nervous system (CNS) tissue of mammals. More recently, sphingolipid LCBs ranging from C18 to C22 alkyl chain lengths in the grey and white matter of human brains were reported [79]. In addition to tissues pertaining to the CNS, a spectrum of uncommon sphingoid bases was also recorded in human plasma samples [49].

With the advent of targeted metabolomics and more granular detection methods, scientists can now appreciate and record these atypical S1P variants in mammals. For example, elevated levels of d16:1 S1P were identified in human cancer samples [78]. This can have far-reaching implications for the chemical ability of these atypical S1P moieties to engage downstream signaling via G protein-coupled receptors [73]. Recently, pioneering work by Muralidharan et al. has provided a comprehensive reference map of over 100 sphingolipid species in 21 murine tissues [42]. Using a targeted metabolomics approach, the group could detect d16:1 S1P in plasma, brown adipose tissue (BAT) and muscles. High levels of d16:1 sphinganine were observed in the skin [42]. In the case of d20:1 S1P, they have also revealed significant levels in the brain and to a lesser extent in the stomach, muscle and intestine [42]. Previous work from our group corroborates the expression of atypical S1P metabolites with varying alkyl chain lengths in both mouse and human, in samples obtained from the CNS as well as several peripheral organs [17, 77].

## The synthesis of atypical sphingolipids

The de novo biosynthesis of all sphingolipids is initiated by the enzyme serine palmitoyltransferase (SPT), located in the smooth endoplasmic reticulum. SPT serves as both the initiator and rate limiting factor in this pathway by catalyzing the reaction of its preferred substrates palmitoyl-CoA and serine to 3-ketosphinganine. This is then reduced to sphinganine in a NADPH-dependent manner, from which ceramide is formed after acylation and dehydration [35]. An alternative possibility of ceramide formation is the hydrolytic release from sphingomyelin by a sphingomyelinase. A ceramidase catalyzes the deacylation to sphingosine, which in turn is phosphorylated to S1P by two subtypes of sphingosine kinases (SPHK1 or SPHK2). S1P can be dephosphorylated back to sphingosine by a S1P phosphatase or irreversibly degraded to hexadecenal and phosphoethanolamine by S1P lyase [23, 35].

Most commonly, the LCBs are derived from the initial condensation of a fatty acyl CoA with the amino acid serine, mediated by the enzyme SPT. Under homeostasis, palmitoyl-CoA (C16-carbon chain) is the preferred fatty acid used by the enzyme SPT, generating a C18 LCB, which is the most prevalent sphingolipid alkyl chain length found in eukaryotes. Nonetheless, SPT also catalyzes the condensation of myristoyl-CoA (C14-carbon chain) or stearoyl-CoA (C18-carbon chain) to produce C16 or C20 LCBs, respectively. Recently, several studies confirmed the detection of such atypical sphingolipid chain lengths in both humans and mice [14].

**Fig. 1:**
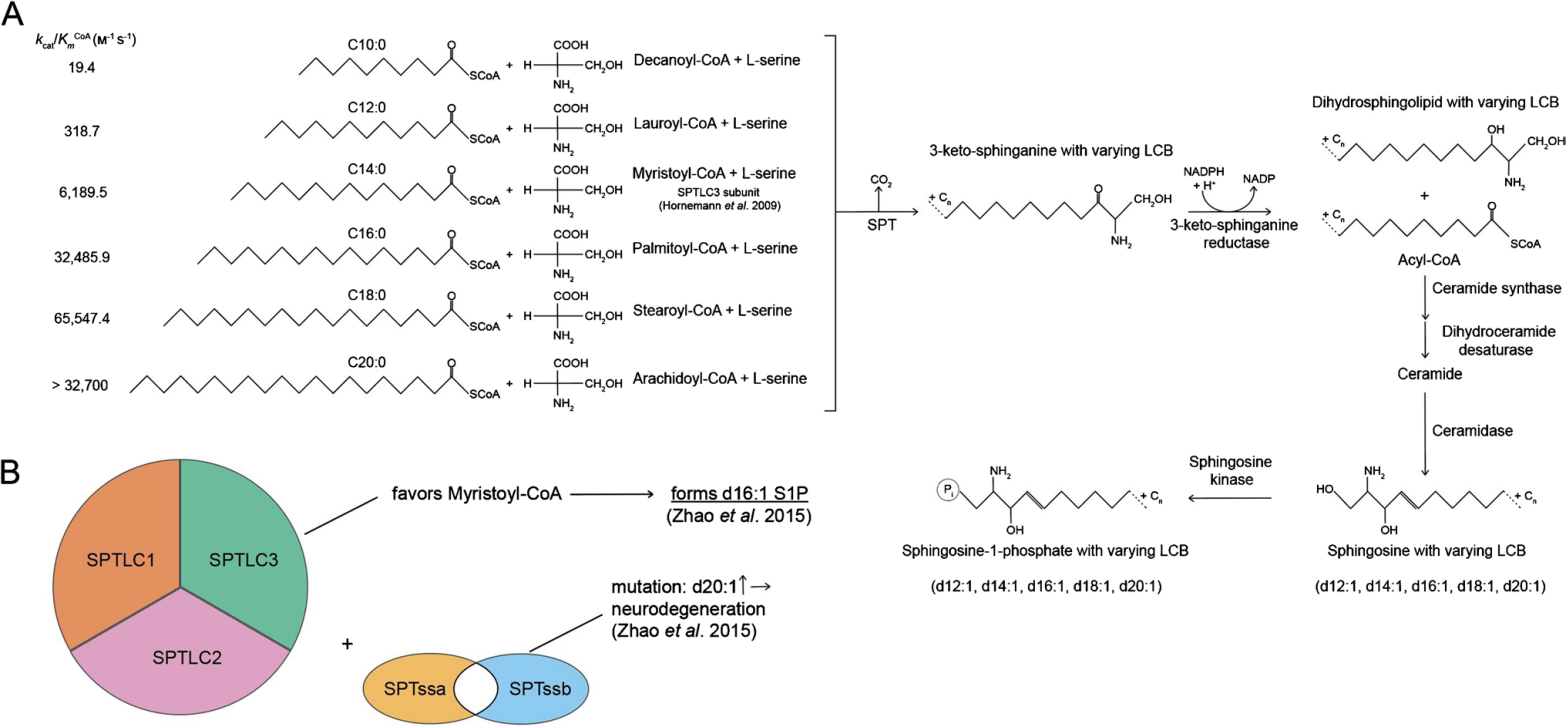
**A** Biosynthesis of atypical sphingosine-1-phosphate metabolites. De novo sphingolipid biosynthesis is initiated in the smooth endoplasmic reticulum. Here the α-aminocarbonic acid serine and the lipids decanoyl-CoA, lauroyl-CoA, myristoyl-CoA, palmitoyl-CoA, stearoyl-CoA, and arachidoyl-CoA are enzymatically processed by the key enzyme serine palmitoyltransferase (SPT) to 3-keto-sphinganine with varying LCBs. The further enzymatic reactions include a reduction to dihydrosphingolipid with varying LCBs, followed by a synthase reaction to dihydroceramide and a desaturase reaction to ceramide, followed by a deacylation by ceramidase to form sphingosine. Sphingosine is phosphorylated by sphingosine kinases to S1P with varying LCBs. Enzymatic activities of SPT for these metabolites were taken from Raman et al. [56]. **B** The enzyme SPT is composed of two large subunits (SPTLC1 and SPTLC2 or SPTLC3) and one small subunit (SPTssa or SPTssb). The subunit composition of SPT dictates the fatty-acyl-CoA substrates utilized. SPTLC1/3/SPTssa can use myristoyl-CoA, resulting in d16 bases. A SPTssb mutation increases the affinity of SPT towards C18 acyl-CoA substrates and significantly elevates C20 LCB production in the mutant brain and eye (Zhao et al., 2015). K_d_- Dissociation constant

The enzyme SPT is composed of two large subunits (SPTLC1 and SPTLC2 or SPTLC3) and one small subunit (SPTssa or SPTssb). These subunits enhance the activity of the core SPT complex and determine the overall SPT activity [20]. Moreover, the subunit composition of SPT dictates the fatty acyl CoA substrates utilized. The complex of SPTLC1/2/ssSPTa primarily processes palmitoyl-CoA, resulting in the production of the most abundant C18 sphingolipids. However, depending on the combination of SPTLC3 and/or SPTssb, shorter or longer acyl-CoAs such as myristoyl-CoA (14:0) or stearoyl-CoA (18:0) can be utilized, leading to the production of less common sphingoid bases. For instance, SPTLC1/3/SPTssa can use myristoyl-CoA equally to palmitoyl-CoA, resulting in d16 bases, while SPTLC1/3/SPTssb can utilize myristoyl- or stearoyl-CoA (Fig. 1) [27]. Notably, SPT activity is inversely regulated in response to the concentration of sphingolipids [66]. This regulatory mechanism involves ORM1-like proteins (ORMDLs), membrane-bound proteins located in the endoplasmic reticulum which form stable complexes with SPT [13]. The brain-specific activity of SPT in rodents is enhanced after birth but subsequently declines despite a concurrent rise in sphingolipid synthesis during this period. Analysis of the major SPT subunits’ protein levels showed, that SPTLC2 levels closely mirrored the SPT activity pattern, whereas SPTLC1 levels remained relatively constant. Furthermore, Davis et al. revealed that SPTLC3 has the capability to replace SPTLC2 within the core SPT complex [14]. Notably, both protein and mRNA levels of SPTLC3 reached a peak at days 12 and 15 after birth of rats, indicating a shift in the SPT complex composition during the critical phase of myelination [14]. Another intriguing observation was the reciprocal alteration in levels of the small SPT subunits SPTssa and ssb. Specifically, SPTssa mRNA levels experienced a significant decrease during the myelination phase, while the levels of SPTssb increased [14].

In isolated rat oligodendrocytes, a consistent increase in the levels of SPTLC1 and SPTLC2 throughout the differentiation from immature progenitor cells to mature myelinating oligodendrocytes was reported [24, 45].

### Sphingolipid pathways generating atypical S1P chain lengths

S1P generation in cells is followed by a ceramide synthesis, a central step in the sphingolipid metabolism. Ceramides are pooled by de novo synthesis, degradation of sphingomyelins, catabolism of sphingosine and hydrolysis of glucosyl or lactosyl ceramides or from the breakdown of ceramide-1-phosphate [13]. To date, it is not known whether sphingolipid species of different chain lengths are primarily synthesized de novo or recycled within the salvage pathway and how the balance of these products shifts under homeostasis and pathological conditions.

In general, changes in the de novo synthesis of sphingolipid metabolites can only be compensated within a narrow margin, which may explain their role in determining cell fate concerning survival and apoptosis, the so called “sphingolipid rheostat” [12, 28]. The central product ceramide is [45] converted into sphingomyelin and subsequently into S1P. Further conversion to complex sphingolipids can be considered an important detoxification process [57]. Saturation of the alkyl chains occurs rapidly and therefore, it has been suggested that the generation of sphingoid bases from the salvage pathway represents a negative control mechanism [44]. Exogenously applied sphingolipid metabolites can also be processed via the de novo pathway or function as negative regulators. It was observed that radioactively labelled sphingosine is converted into glycosphingolipids depending on its chain length, whereby the conversion was significantly more pronounced with substrates of shorter chain lengths [15]. However, the N-acylation efficiency for sphingosine to ceramides seems to differ depending on the cell type studied. For example, cerebellar astrocytes appear to be much better equipped for this than cerebellar neurons [57]. Exogenous supplementation studies have corroborated the vital importance of de novo sphingolipid synthesis. For example, it is known that an oversupply of nutritive fatty acids leads to increased de novo sphingolipid synthesis [31]. It has also been shown that enriching the diet with certain fatty acids can significantly influence the lipid balance, whereby a diet containing stearic acid is associated with vasoprotection [32]. In contrast, enriching the diet with myristic acid confers proatherosclerotic effects [19]. Studies on radioactively labelled palmitic or stearic acid confirmed their swift uptake into rat cerebellar granule cells in vitro to be used for de novo sphingolipid synthesis. After treatment with labelled palmitic acid, an almost 100-fold higher proportion of radioactive metabolites was found than after treatment with labelled stearic acid [9]. In addition, palmitic acid appeared to be completely incorporated into C18 sphingoid bases, whereas derivatives of radiolabelled stearic acid were found in both C18 and C20 sphingolipids [81]. This indicates that a small part of the substrate is oxidized and the resulting acetyl-CoA would be made available for the synthesis of C18 sphingolipids [9]. When interpreting these data, however, it should be noted that the extremely low and, in some cases, lacking detection of C20 sphingolipids could be due to the prevailing technical limitations at the time.

Furthermore, the production of sphingoid bases depends not only on the presence of the corresponding substrates but also on enzyme availability or their isoforms involved where homeostatic conditions can significantly deviate from diseased conditions. For example, it has been shown that the function of SPT and in particular that of the SPTLC2 subunit can be influenced both at the expression (e.g., by cytokines or radiation) and at the activity level (e.g., by etoposide or heat shock proteins) [26]. Treatment of Western diet–fed mice with the SPT inhibitor myriocin led not only to a reduction in sphingolipid species but also in cholesterol and triglycerides and to a shift in the lipoprotein ratio in favor of HDL cholesterol and to a consecutive reduction of atherosclerotic plaques [53]. Conversely, ethanol-induced neuronal degeneration was shown to coincide with SPT activation and subsequent caspase-3 induction [60]. Steroids are also able to induce SPTLC2 as well as various ceramidases. Therefore, they not only engage in transcriptional regulation but can also confer post-translational regulation by forming inhibitory end products [26]. In the case of an accumulation of free sphinganine which is usually only present in trace amounts [68], by inhibition of ceramide synthases, a preferential synthesis of sphingomyelin at the expense of complex sphingolipids was observed [39]. Conditions associated with impaired vesicular transport can also produce a shift in favor of certain sphingolipid species [57].

The data on the role of the salvage pathway in relation to the regeneration of sphingoid bases of different chain lengths is even sparser than of the de novo synthesis. However, Schiffmann et al. were able to report that the cyclooxygenase (COX)2 inhibitor celecoxib exerted its toxic effects on hepatocytes partly via a specific induction of C16-S1P, which could be attributed to an induction of ceramide synthase 6 (CERS6) [62]. Nevertheless, further evaluation of this process revealed that the C16 sphingoid bases appeared to originate primarily from the salvage pathway [62].

To date, several reports have confirmed the increase of sphingolipids from both the de novo and salvage pathways when mammalian cells were exposed to chemotherapeutic drugs, heat stress, or apoptotic stimuli such as Fas ligands or tumor necrosis factor. These sphingolipids have the potential to influence inflammation, stress response, apoptosis and the cell cycle. Thus, the production of signaling molecules via de novo synthesis and/or salvage pathways is crucial not only for maintaining homeostasis but also for accumulation or depletion of atypical sphingolipids with varying LCBs [33].

## S1P signaling and current therapeutic perspectives of atypical S1P chain lengths

Sphingosine is phosphorylated to S1P by two sphingosine kinase isoforms (SPHK1 and SPHK2). The earliest reports regarded S1P merely as an intermediate product of the sphingolipid pathway [40] until the discovery of its contribution to cell growth and apoptosis [47, 48]. S1P represents a minor fraction of the total sphingolipids, but can act as an intracellular second messenger as well as signal via its five identified G-protein coupled receptors S1P_1_ to S1P_5_ [28, 35]. In mammals, S1P receptors are ubiquitously but differentially expressed depending on the cell type and biological response such as cardiovascular development [3, 16, 72], neural cell proliferation [54, 67, 80], immune cell migration [52], inflammation [82], fibrosis [29, 64] and endothelial function [30, 55]. Until now, there are few investigations that have elucidated the physiological role, mechanisms, and S1P receptor activation by the less abundant atypical sphingolipids d16:1 and d20:1 S1Ps.

A recent study revealed that S1P with acyl chain lengths ranging from C16 to C20 differentially engaged its downstream S1P receptors. This study could directly link ligand efficacy of the evoked downstream receptor signal to the alkyl chain length [73]. The authors concluded that the receptor activation depended on the hydrophobic interactions between the lipid tail of the ligand and the residues at a distinct binding site located between the receptors’ transmembrane (TM) α-helices [73].

Several S1P receptor (S1P_R_) modulators such as fingolimod, siponimod and ponesimod are FDA approved drugs to treat MS [11]. The new-generation S1P_R_ drugs such as ozanimod and estrasimod have shown beneficial effects in inflammatory bowel disease, whereas amiselimod and cenerimod have been tested in human patients with systemic lupus erythematosus (SLE). Also, ponesimod has demonstrated beneficial results in clinical trials performed on psoriasis patients. In addition, there are novel drugs under development that target S1P transport, generation, or degradation in the context of renal diseases, stroke, amyotrophic lateral sclerosis (ALS), endometriosis, glioblastoma, macular degeneration, asthma and biliary cholangitis [8, 70, 74].

Recently, higher concentrations of d16:1 S1P were detected in human cancer patients undergoing chemotherapy with oxaliplatin [78]. Interestingly, decreased levels of d16:1 S1P in plasma of patients with vascular dementia were revealed and proposed a potential role in tuning d18:1 S1P-mediated cytokine production. It was described that overexpression of the SPT subunit SPTLC3 leads to the production of sphingolipids with a C16 sphingoid base [10]. The atypical sphingolipid with a C16 base d16:1 S1P, was recently shown to mediate connective tissue growth factor (CTGF) induction via S1P_2_ in renal cell carcinoma (RCC). d16:1 S1P versus d18:1 and d20:1 S1P led to the highest CTGF induction in RCC cells (A498) via S1P_2_. Accordingly, d16:1 S1, and CTGF levels were elevated in RCC as compared to adjacent healthy tissue, suggesting that elevated plasma levels of d16:1 S1P may play a procarcinogenic role in the development of RCC via the induction of CTGF [17].

On the other hand, C20-LCBs have been reported to be abundantly expressed in gangliosides of different mammalian species [9]. Furthermore, elevated concentrations of sphingolipids with C20-LCBs in plasma have been shown to be a predictive risk factor concerning cardiovascular events [32]. The relevance of C16 and C20 sphingosine and DHS was affirmed after their isolation from the spinal cords of an experimental autoimmune encephalitis (EAE) model of MS in rabbits [65]. In 2008, the grey matter sphingolipid content of human MS patients showed decreased levels of C20 sphingomyelins only in patients with active MS, whereas C16 sphingolipids declined in the grey matter of both active and inactive MS patients [79]. In a detailed review, brain functionality and neurodegenerative processes were reported to depend on the ratio between C18- and C20-gangliosides [68]. In addition to the CNS milieu, atypical sphingoid bases, especially C20 chain lengths, were found to be elevated and therefore regarded as potential biomarkers for cardiovascular events [49]. Likewise, the occurrence of d16:0 and d16:1 sphingolipids in the myocardium was reported [59], further supporting the occurrence of atypical sphingolipids in different organs in mammals.

The first study to unveil the role of long chain S1P (d20:1) reported that elevation of sphingolipids containing C20 LCBs has detrimental neurodegenerative effects in the brain and the retina [83]. The group identified a *Sptssb* mutation, *Stellar* (Stl), which increased the affinity of the SPT towards C18 acyl-CoA substrates by twofold and significantly elevated C20 LCB production in the mutant murine brain and eye. These findings indicate that the composition of the SPT complex changes regarding both major and minor subunits and thereby influences the levels of sphingolipids of different chain lengths.

**Fig. 2:**
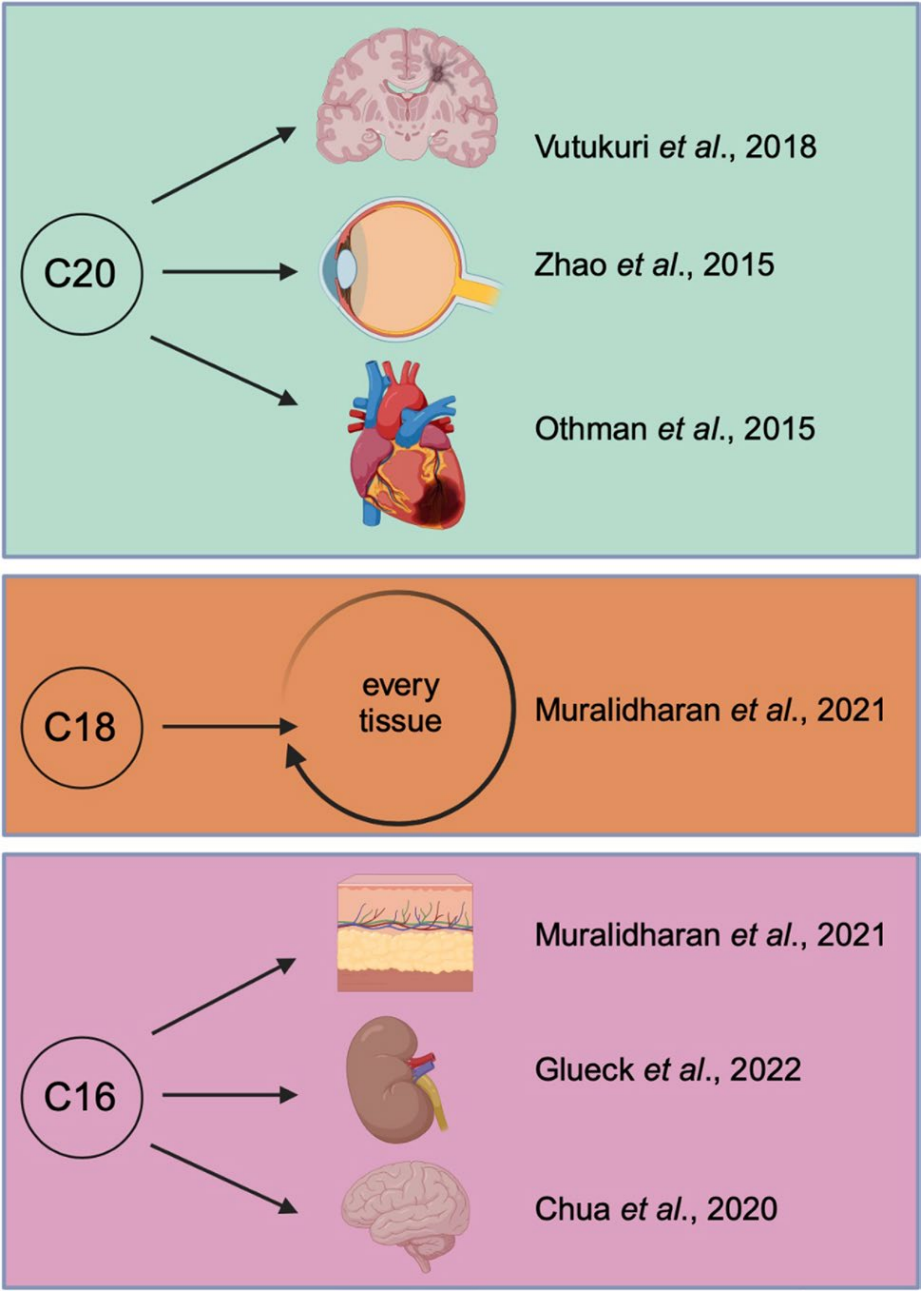
Identification of atypical sphingosine-1-phosphate metabolites in several organs and their implications in health and diseases. Reduced levels of d20:1 have been reported in whole brain homogenate and mouse brain microvessels in a septic encephalopathy mouse model (Vutukuri et al., 2018). It was also shown that significantly elevated C20 LCBs in the mutant mouse brain and eye play a detrimental role resulting in neurodegenerative effects (Zhao et al., 2015). Elevated C20 LCBs have been regarded as predictive biomarkers of cardiovascular events independently from conventional cardiovascular risk factors (Othman et al., 2015). The new atlas of murine sphingolipids displayed that d18:1 sphingosine (SPH) is the most abundant mammalian SPH and showed a very characteristic peak value in the skin for d16:1 SPH (Muralidharan et al., 2021). Glueck et al. confirmed the levels of C16:1 in human kidney samples and investigated the role of d16:1 S1P in renal cell carcinoma (RCC) Glueck et al. [17]. Reduced levels of d16:1 have also been shown in vascular cognitive impairment Chua et al. [10]

Concerning acute stroke, alterations of the sphingolipidome in the peri-infarct tissue during hemorrhagic transformation (HT) were also previously observed [37]. Here, ischemic stroke resulted in reduced S1P whilst ceramides were elevated 6 h post-ischemia. Apart from C18 sphinganine, the topmost expression levels of all other immediate S1P relatives and S1P itself were qualitatively linked to a more frequent conversion towards HT [37]. The coinciding disruption of the blood–brain barrier selectivity may therefore allow such markers to be detectable in the plasma where they could serve as biomarkers. For example, plasma C20-sphingolipid levels have been shown to be predictive biomarkers for cardiovascular events, even after adjusting for major cardiovascular risk factors, medication and coronary artery disease (Fig. 2) [49].

## Future therapeutic perspectives of atypical S1P mediators

To date, the influence of drugs targeting the S1P-S1P_R_ axis on levels of S1P mediators with atypical chain lengths remains yet to be investigated. The levels of atypical S1P chain lengths in plasma and organs are several times lower than the abundant d18:1 S1P. It has already been shown that the alkyl chain length influences the degree of S1P_R_ activation [73]. Even though there is evidence that interferon (IFN)-ß increases the plasma levels of C16:0, C18:0, C20:0, and C24:1 ceramides in MS patients [50], to our knowledge, there is no data on how MS drugs could affect the synthesis and signaling of sphingolipids with varying alkyl chain lengths. In addition, it remains unknown, if these sphingolipids act as conflicting chemotactic agents for immune cell egress. The egress of lymphocytes and the vascular barrier functions depends upon the ability of the lymphocytes and endothelial cells to sense surrounding S1P levels. S1P triggers lymphocytes to egress via chemotaxis from lymphoid organs where low S1P levels are established (low nanomolar) into the circulation where higher S1P levels exist (high nanomolar) [36]. It has been shown that a failure to sustain these high and low levels of S1P in the circulation and tissues, respectively, leads to lymphopenia, compromised barrier function and other tissue dysfunctions [46]. In fact, the induced down-regulation of S1P receptors by S1P modulators used for autoimmune diseases such as MS, exploits this reduced functional activity towards S1P upon S1P receptor downregulation. Ex vivo it has been shown that an incubation with 1 nM of d18:1 S1P for 20 min is sufficient to internalize the S1P_1_ receptor on lymphocytes [10]. Although the total d18:1 S1P in plasma is in low micromolar concentrations, Schwab and Cyster have provided evidence that the bioactive d18:1 S1P in plasma is ~ 10 nM which is nearly the *K*_d_ of d18:1 S1P to activate its cognate receptors [63]. The levels of d16:1 and d20:1 S1Ps are in low nanomolar range in comparison to d18:1 in different organs as well as in plasma [17, 76, 77], but can activate S1P receptors at similar levels [73]. The stability of d18:1 S1P in plasma has been shown to be ~ 15 min [75], whereas for other chain lengths, this has not been validated yet. As the levels of atypical S1P chain lengths are nearly sufficient to trigger lymphocyte activation, it will be interesting to investigate their contribution to the lymphoid organ egress (e.g., from the spleen) of lymphocytes and other immune cells and how the immune cells sense atypical S1P moieties and the induced action on different S1P_R_. Recently, Guo et al*.* have identified ApoM/S1P signaling to promote cardiac protection in anthracycline-mediated cardiotoxicity via regulating autophagy and lysosomal degradation [18]. In humans, patients with anthracycline-induced cardiomyopathy showed reduced levels of circulating ApoM. In case of elevated ApoM levels, myocardial autophagy was prevented. These reports confirm the protective effects of the ApoM/S1P axis [18]. In the circulation, d18:1 S1P binds 70% to ApoM and the remaining S1P to albumin [4]. As the bioavailability of S1P is determined by the degree of the binding of S1P to its chaperones, it will be interesting to determine the binding pattern of atypical S1P moieties and how this pattern regulates features of tissue/cellular inflammation and vascular permeability as has been ascertained for d18:1 S1P. Research on atypical S1P chain lengths is rapidly progressing, and further understanding on their accrual, intracellular metabolism, secretion and effect on different S1P receptors is important as it will enable us to untangle the controversies concerning the biological implications of S1P signaling (Table 1). To address these questions, recent advances like the synthesis of optical S1Ps and click S1Ps will be invaluable. In 2019, Morstein et al. have generated photoswitchable S1P and sphingosine and termed them “PhotoS1P” and “PhotoSphingosine” (“PhotoSph”), which they demonstrated to optically control S1P_1-3_ receptors both in vitro and in vivo [41]. Using PhotoS1P, the group successfully displayed a reversible modulation of S1P_3_-dependent pain hypersensitivity in mice. In addition, using lipid mass spectrometry, they could create a metabolic map of PhotoSph and PhotoS1P, confirming the formation of these photo lipids in a light-dependent manner [41]. Similarly, Sternstein et al*.* have developed clickable S1P derivatives with a terminal azido functional group (S1P-N3). This invention enables intricate studies on the S1P metabolism and enables its intracellular tracking [71]. The authors demonstrated that the transfection of HEK293T cells with S1P_1_-GFP followed by incubation with S1P-N3 resulted in the internalization of the S1P_1_ receptor proving the functionality of the click S1P substrate to study receptor activation. Furthermore, in U20s osteosarcoma cells, the click S1P dye conjugate with DBCO-BODIPY was shown to be predominantly distributed at the nuclear membrane and in the endoplasmic reticulum. Accordingly, these novel tools are immensely useful to study the metabolism of S1P [71]. These emerging technologies should be employed to test the distribution of different S1P chain lengths and their action on S1P receptors which could pave way for fine tuning S1P-based therapeutics.

**Table 1:** Literature on different S1P chain lengths and implications in health and diseases

Author	Organ/cell line	Findings
Zhao et al. [83]	Brain	Mutation in the Sptssb gene leads to an increase in the production of 20-carbon (C20) LCBs in the mouse brain and eye, leading to neurodegenerative effects
Chigorin et al. [9]	Brain	Confirmed C20:1 sphingosine incorporation in rat cerebellar granular cells
Vutukuri et al. [76]	Brain	In septic encephalopathy model detected reduced levels of d18:1 as well as d20:1 S1Ps in whole brain homogenate and mouse brain microvessels
Vutukuri et al. [77]	Brain	Confirmed the detection of d20:1 S1P in mice CNS and human glioblastoma
Sonnino et al. [68]	CNS	Hypothesized the role of C18- and C20-sphingosine in gangliosides which modulate membrane domain organization and cell properties during CNS development and aging
Nagree et al. [43]	Spinal cord	Mutations in ASAH1 results in elevated levels of d20:1 S1P in neurodegeneration
Othman et al. [49]	Plasma	Demonstrated elevated plasma C20 sphingoid bases as novel biomarkers in cardiovascular disease
Chua et al. [10]	Plasma	Analyzed human plasma samples and identified peripheral S1Ps, specifically d18:1 to d16:1 ratio as potential biomarker for vascular cognitive impairment (VCI)
Muralidharan et al. [42]	Different organs	Provided a new atlas of murine sphingolipids across 21 tissues in C57BL/6 mice. They ascertained the presence of different S1P chain lengths, revealed tissue- and sex-specific S1P distributions, providing a detailed sphingolipidomic map for health and disease research
Glueck et al. [17]	Kidney	Confirmed the levels of d16:1 in various mouse tissues and human kidney samples and investigated the role of d16:1 S1P in renal cell carcinoma (RCC)
Troupiotis-Tsailaki et al. [73]	CHO-K1 cells	Performed in vitro molecular dynamic simulations and functional assays and confirmed that the efficacy of sphingosine-1-phosphate (S1P) analogues is influenced by the length of their alkyl chains, impacting their interaction with different S1P receptors

## Conclusion

Atypical S1P chain lengths exhibit distinct biological activities compared to the widely studied d18:1 S1P. Previous research has shown that S1P_R_ have a differential binding affinity and selectivity to these atypical S1P molecules. Accordingly, signaling strength and/or functional outcome upon receptor binding can be heavily affected and maybe account for disease pathology. Exploring the potential of atypical S1P species could represent a promising frontier in therapeutic development especially to selectively modulate S1P_R_ signaling. Ultimately, this could open new avenues for treatment of a wide range of diseases, including cardiovascular disorders, cancer, autoimmunity and fibrotic diseases.

## Data Availability

No datasets were generated or analysed during the current study.
